# Supplementary material for: DDC-Promoter-Driven Chemogenetic Activation of SNpc Dopaminergic Neurons Alleviates Parkinsonian Motor Symptoms
Source: Int J Mol Sci. 2023 Jan 27;24(3):2491. doi: 10.3390/ijms24032491 (PMC9916413; doi:10.3390/ijms24032491)
Supplement: Supplementary file 1 [file ijms-24-02491-s001.zip › DDC supple IJMS revison final track on.pdf]

Supplementary Materials

DDC-Promoter-Driven Chemogenetic Activation of SNpc Dopaminergic Neurons Alleviates Parkinsonian Motor Symptoms

Dong-Chan Seo [1,2,3], Yeon Ha Ju [2], Jin-Ju Seo [1,4], Soo-Jin Oh [2], C. Justin Lee [5], Seung Eun Lee [1,*] and Min-Ho Nam [2,6,*]

1 Research Animal Resource Center, Korea Institute of Science and Technology (KIST), Seoul 02456, Republic of Korea
2 Brain Science Institute, Korea Institute of Science and Technology (KIST), Seoul 02456, Republic of Korea
3 Department of Integrated Biomedical and Life Sciences, College of Health Science, Korea University, Seoul 02841, Republic of Korea
4 Technological Convergence Center, Korea Institute of Science and Technology (KIST), Seoul 02456, Republic of Korea
5 Center for Cognition and Sociality, Institute for Basic Science, Daejeon 34126, Republic of Korea
6 Department of KHU-KIST Convergence Science & Technology, Kyung Hee University, Seoul 02447, Republic of Korea
* Correspondence: selee@kist.re.kr (S.E.L.); dr.namminho@kist.re.kr (M.-H.N.)

Sample no.	#1	#2	#3	#4	avg.
Number of rDDC-mCh$^+$ neurons	171	189	178	101	160
Number of rDDC-mCh$^+$DDC$^+$ neurons	133	141	123	75	118
rDDC-mCh$^+$DDC$^+$/rDDC-mCh$^+$ (%)	77.78%	74.60%	69.10%	74.26%	73.93%

Table S1. Raw data table for Figure 1e.

	sAP frequency (Hz)	
Rat no.	Before	+ CNO
#1	1.2666	2.0666
#2	11.1333	20.6667
#3	7.5333	9.1333

#4	12.4333	17.1
#5	6.36	15.1

Table S2. Raw data table for Figure 2f.

Stepping test		Stepping ratio (contralateral step number / total step number)		
Group	Rat no.	Before	PD	+ CNO
Control	#1	0.500	0.449	0.443
	#2	0.482	0.429	0.417
	#3	0.500	0.438	0.400
	#4	0.510	0.460	0.440
	#5	0.480	0.430	0.460
	#6	0.500	0.440	0.390
hM3Dq	#1	0.500	0.418	0.464
	#2	0.506	0.424	0.508
	#3	0.490	0.450	0.500
	#4	0.500	0.510	0.490
	#5	0.490	0.450	0.480
	#6	0.490	0.450	0.490

Table S3. Raw data table for Figure 3c.

Balance beam test		Foot slip (#)		
Group	Rat no.	Before	PD	+ CNO
Control	#1	2.5	3	5.5
	#2	1	2	2.5
	#3	2	5.5	6
	#4	0.5	2.5	8
	#5	2.5	1.5	8.5
	#6	1	3.5	6
hM3Dq	#1	5.5	4.5	#1
	#2	0.5	2.5	2
	#3	2.5	6.5	1
	#4	2	4.5	4.5
	#5	1.5	5	0.5

#6	1	7.5	2.5

Table S4. Raw data table for Figure 4b.

Balance beam test		Balance beam score		
Group	Rat no.	Before	PD	+ CNO
Control	#1	3.5	3.5	2.5
	#2	4.5	4	3.5
	#3	3.5	2.5	2.5
	#4	4.5	3.5	1.5
	#5	4.5	3.5	2.5
	#6	4.5	2	3
hM3Dq	#1	4.5	2.5	3
	#2	4.5	3.5	4
	#3	3.5	2.5	4
	#4	4	2	2.5
	#5	4	2.5	4.5
	#6	4.5	2	4

Table S5. Raw data table for Figure 4c.

Group	Rat no.	Portion of c-fos^{+} out of mCh^{+} neurons (%)
Control + CNO	#1	0%
	#2	6%
	#3	14%
	#4	5%
	#5	13%
	#6	17%
	#7	35%
	#8	44%
hM3Dq + CNO	#1	80%
	#2	70%
	#3	75%

#4	77%
#5	61%
#6	46%
#7	68%
#8	70%

Table S6. Raw data table for Figure 5b.

		TH+ neuron ratio (ipsi/contra) (%)			
Group	Rat no.	AP -5.30 ~ 5.59 mm	AP -5.30 ~ 5.79 mm	AP -5.80 ~ 6.03 mm	AP -6.04 ~ 6.30 mm
Control + CNO	#1	37.85%	31.35%	2.3%	62.09%
	#2	50.76%	30.96%	9.5%	48.59%
	#3	47.16%	32.21%	53.44%	13.08%
	#4	41.7%	27.27%	81.56%	70%
	#5	40.55%	17.62%	16.08%	54.76%
	#6	64.68%	8%	41.33%	73.61%
	#7	33.86%	23.75%	74.23%	73.79%
	#8	54.31%	35.88%	50.56%	77.61%
hM3Dq + CNO	#1	60.54%	95.77%	98%	61.33%
	#2	88.67%	44%	90%	63.72%
	#3	76.43%	41.18%	90.28%	89.62%
	#4	82.28%	68.29%	96.4%	88.09%
	#5	80.47%	72.31%	45.93%	87.3%
	#6	84.73%	75.92%	65.42%	26.24%
	#7	53.55%	65.42%	97.99%	76.5%
	#8	61.94%	80.53%	91.67%	71.63%

Table S7. Raw data table for Figure 6b,c.

Group	slice	% of striatal TH staining density (ipsi/contra)
Control + CNO	#1	30%
	#2	48%
	#3	46%
	#4	49%
	#5	58%
	#6	57%
	#7	68%
	#8	54%
hM3Dq + CNO	#1	74%
	#2	72%
	#3	90%
	#4	72%
	#5	90%
	#6	81%
	#7	72%
	#8	86%

Table S8. Raw data table for Figure 7b.
